# miRNA-7062-5p Promoting Bone Resorption After Bone Metastasis of Colorectal Cancer Through Inhibiting GPR65

**DOI:** 10.3389/fcell.2021.681968

**Published:** 2021-08-17

**Authors:** Liang Chen, Yu Wang, Xingchen Lu, Lili Zhang, Ziming Wang

**Affiliations:** ^1^Department of Orthopedics, Army Medical Center, Army Medical University, Chongqing, China; ^2^Department of Military Psychology, College of Psychology, Army Medical University, Chongqing, China

**Keywords:** miRNA-7062-5p, GPR65, colorectal cancer, bone metastasis, osteoclast

## Abstract

Bone metastasis is positively associated with a poor prognosis in patients with colorectal cancer (CRC). CRC always leads to osteolytic change, which is regulated by aberrant activation of osteoclasts. MicroRNAs are remarkedly involved in metastasis of CRC; however, their role in bone metastasis of CRC is still unclear. The aim of this study is to find key microRNAs that are critical to bone resorption in bone metastasis of CRC. In this study, bone metastasis model was established through intratibially injecting CT-26 cells or MC-38 cells. Tartrate-resistant acid phosphatase (TRAP) staining was performed to explore the osteoclastogenesis of primary early osteoclast precursors (OCPs) after stimulation by CT-26 conditioned medium (CM). Then, microarray assay was performed to find differentially expressed miRNAs and mRNAs. The target gene of miRNA was confirmed by dual-luciferase analysis. The effect of miRNA, its target gene on osteoclastogenesis, and involved pathways were explored by Western blot, immunofluorescence analysis, and TRAP staining. Finally, the effect of miRNA on bone resorption *in vivo* was observed. miRNA-7062-5p was upregulated in early OCPs cultured in CT-26 CM or MC-38 CM. GPR65 was proven to be the target gene of miRNA-7062-5p. Overexpression of GPR65 can rescue the osteoclastogenesis caused by miRNA-7062-5p through activation of AMPK pathway. Local injection of miRNA-7062-5p inhibitors efficiently improved the bone resorption. Our study found the role of miRNA-7062-5p in regulating osteoclast formation, and our findings provided a potential therapeutic target in treatment of bone metastasis of CRC.

## Introduction

Although the incidence of bone metastasis from colorectal cancer (CRC) is relatively rare in clinic, the prognosis is pessimistic. Patients with CRC presenting with bone metastasis are often younger and have a poor prognosis; 5-year survival rate was only 5.7% (Kawamura et al., 2018). Moreover, the treatment is insufficient, partly because the underlying mechanism of bone metastasis of CRC is still clearly unexplored.

In bone microenvironment, the balance between osteoblasts and osteoclasts maintains the homeostasis of bone remodeling. CRC is a kind of osteolytic tumors. For these types of tumors, aberrant activation of osteoclasts promotes the bone resorption and pathologic fractures (Clohisy et al., 1996; Li et al., 2019b). Osteoclasts derive from monocytes/macrophage lineage. RANKL determines monocytes/macrophages to commit to osteoclast precursors (OCPs) (Liang et al., 2019). In tumor microenvironment, cancer cells can directly promote the osteoclastogenesis of OCPs through RANKL-dependent or independent ways (Liang et al., 2019). However, little is known on how the osteoclasts or its precursors change in CRC microenvironment.

MicroRNAs remarkedly participate in the progression and metastasis of CRC and are important regulators for osteoclastogenesis. Some microRNAs, such as miRNA-802, miRNA-875-3p, miRNA-574, miRNA-708, miRNA-206, and miRNA-27b, can attenuate the progression of CRC, whereas miRNA-503 facilitates the tumor growth (Ye et al., 2013; Park et al., 2018; Li et al., 2019a, 2020b; Sun et al., 2019; Zhang et al., 2020). In addition, miRNAs can also be used to predict the prognosis of CRC. It was also reported that miRNA-483, miRNA-218, miRNA-199a-5p, miRNA-133a, and miRNA-340 can regulate the osteoclast formation (Zhao et al., 2017; Guo et al., 2018, 2019; Li et al., 2018, 2020a). Considering the importance of miRNAs in both tumor progression and osteoclastogenesis, it is worthy to know the function of miRNAs in bone metastasis of CRC.

In this study, we identified that miRNA-7062-5p was treated by condional medium collected from CRC and promoted the osteoclastogenesis through targeting GPR65. Downregulating miRNA-7062-5p efficiently attenuated the progression of bone destruction caused by CRC.

## Materials and Methods

### Animals

All procedures involving mice and experimental protocols were approved by the Institutional Animal Care and Use Committee of Daping Hospital at Army Medical University. C57BL/6 or BALB/c mice for all experiments were 6–8 weeks of age. CT-26 and MC-38 cells are two commonly used CRC cell lines. Thus, for bone metastasis studies, 500,000 CT-26 or MC-38 cells were resuspended in phosphate-buffered saline (PBS) and injected into tibias of mice after anesthetization. Antagomir-7062-5p or scrambled controls dissolved in PBS (10 μg/50 μL) were injected intravenously every 3 days for continuous 3 weeks. At specific timepoints, mice were killed, and the hindlimbs were removed and fixed in 4% paraformaldehyde for histochemical staining or tartrate-resistant acid phosphatase (TRAP) staining. Five to 10 mice were used per group.

### Histological Analysis

Tibias were dissected and fixed for 48 h. Then, the samples were decalcified by daily change of 15% tetrasodium EDTA for 3 weeks and embedded in paraffin. For safranin O and fast green staining, samples were cleared twice in xylene and were rehydrated by passage through an ethanol series following by immersion in PBS. The samples were then stained in 0.1% safranin O solution for 3 min and washed in PBS three times. Next, the samples were immersed in 0.1% fast green solution for 10 s and separated in 1% acetum. The samples were then observed and captured. The TRAP staining was performed according to the manufacturers’ instructions (BestBio Biotechnology, Beijing, China). For immunofluorescence analysis, early OCPs were seeded on coverslips and transfected by miRNAs. Then, the samples were fixed in 4% paraformaldehyde for 15 min in 4°C. After paraformaldehyde was discarded, the samples were washed in PBS and incubated in blocking buffer containing 10% donkey serum and 5% bovine serum albumin for 1 h in 37°C. Then, primary antibody was incubated overnight in 4°C. Secondary antibody was added after the samples were washed in PBS followed by staining with Hoechst 33342 (1:1,000) for 30 min. Primary antibody used was anti-GPR65 at 1:200 (Biorbyt, Cambridgeshire, United Kingdom). Secondary antibody was donkey anti-rabbit conjugated with Cy3 at 1:200 (Jackson ImmunoResearch Laboratories, Inc., West Grove, PA, United States).

### Primary Early OCP Isolation and Culture

Bone marrows were rinsed out from marrow cavity of tibias and femurs by sterilized PBS. The cell suspension was collected and centrifuged at 500 *g* for 5 min. After discarding the supernatant, erythrocytes were removed. The cells were stained using CD115 antibody conjugated with APC and RANKL antibody conjugated with PE (Biolegend, San Diego, CA, United States) for 1 h in 4°C. Then, the primary early OCPs labeled as CD115-positive and RANKL-negative were sorted out by FACS.

Isolated early OCPs were cultured in Dulbecco modified eagle medium (DMEM) supplemented with 10% fetal bovine serum, 100 U/mL penicillin, and 100 U/mL streptomycin (Gibco, Thermo Fisher Scientific, Waltham, MA, United States) and macrophage colony-stimulating factor (M-CSF) (50 ng/mL) (Biolegend, San Diego, CA, United States). For osteoclastogenic induction, early OCPs were stimulated by M-CSF (50 ng/mL) and RANKL (50 ng/mL) for at least 4–6 days. In some experiments, early OCPs under osteoclastogenic induction were cultured with conditioned medium (CM) from CT-26 for 4 days.

### Transient Transfection

Agomir-7062-5p, antagomir-7062-5p and their scrambled controls, siRNA pool, and pcDNA3.1 (+) plasmid containing GPR65 were synthesized by GenePharma, Co. (Shanghai, China). Transfection was performed using Lipofectamine RNAiMax Reagent (Invitrogen, Thermo Fisher Scientific). The siRNA sequence for AMPKα: 5′- CUAUGAAUGGAAGGUUGUA-3′, 5′-UACAACCUUCCAUUCAUA-3′.

### Luciferase Reporter Assay

293T cells were seeded into 6-well plates. Cells were transfected with pGL3-GPR65 3′ UTR and pRL-TK (Promega, Madison, WI, United States) Renilla luciferase plasmid, as well as wild-type (wt) or mutant (mut) mir-7062-5p or controls using Lipofectamine RNAiMax Reagent following the manufacturer’s instructions. Luciferase assays were conducted using the dual-luciferase reporter assay system (Promega). The values of luminescent signals from firefly luciferase construct were normalized by Renilla luciferase assay.

### Quantitative Real-Time Polymerase Chain Reaction

The total RNA was extracted from cells using TRIzol reagent (Invitrogen, Thermo Fisher Scientific). First-strand complementary DNA was reversed from 1 mg of total RNA using PrimeScript RT reagent Kit (TakaraBio, Tokyo, Japan) according to the manufacturer’s instructions. The stem-loop reverse transcriptase–polymerase chain reaction (RT-PCR) was used for quantification of miRNA-7062-5p. Quantitative real-time PCR was performed using an SYBR Green Premix Ex TaqTMII Kit (TakaraBio, Tokyo, Japan). GAPDH and U6 were used as normalization controls for mRNA and miRNA, respectively. The sequences of the primers are shown as follows: GAPDH (forward: 5′-TGGATTTGGACGCATTGGTC-3′ and reverse: 5′-TTTGCACTGGTACGTGTTGAT-3′), cathepsin K (forward: 5′-CTGGCTGGGGTTATGTCTCAA-3′, and reverse: 5′-GGCTACGTCCTTACACACGAG-3′), MMP-2 (forward: 5′- TGACTTTCTTGGATCGGGTCG-3′, and reverse: 5′-AAGCA CCACATCAGATGACTG-3′), MMP-9 (forward: 5′-TGTACCG CTATGGTTACACTCG-3′, and reverse: 5′-GGCAGGGACAG TTGCTTCT-3′).

### Western Blot

The cells were lysed with RIPA buffer containing protease inhibitors (Beyotime, Shanghai, China). Total proteins were collected and subjected to sodium dodecyl sulfate–polyacrylamide gel electrophoresis and transferred to polyvinylidene difluoride membranes. The membranes were blocked and incubated with primary antibodies. After being washed, the membranes were then incubated with horseradish peroxidase–linked secondary antibodies. The antibodies used were as follows: GPR65 (1:1,000, Biorbyt), JNK (1:2,000; Santa Cruz, Dallas, TX, United States), phosphorylation JNK (1:2,000; Santa Cruz), AMPKα (Abcam, Cambridge, MA, United States), phosphorylation AMPKα(), GAPDH (Abcam), goat anti-rabbit immunoglobulin G (1:5,000; Abcam).

### Microarray Assay

Total RNA was extracted from early OCPs. MiRNAs were isolated and labeled using biotin and detected with Ambion WT Expression Kit. MiRNA and mRNA microarray assays were completed by CNKINGBIO (Beijing, China). The miRNA 4.0 and Clariom D were used for microarray assay. The data sources of non-coding RNA were from Luo et al. (2013). The signal intensity of each spot was calculated by the program R (2.12.1). Spots that passed the criteria were normalized by the invariant set normalization method. Normalized spot intensities were transformed to gene expression log2 ratios between the control group and treatment group using the pairwise *t*-test. The spots with a | log2 ratio| ≥ 0.585 and a *p* < 0.05 were selected for analysis.

### Statistical Analysis

All data are representative of at least three experiments of similar results performed in triplicate unless otherwise indicated. Data are expressed as mean ± SD. One-way analysis of variance was used to determine the significance of difference between results, with ^∗^*p* < 0.05 being regarded as significant.

## Results

### Secreta Derived From CRC Cells Induce Osteoclastogenesis

**FIGURE 1:**
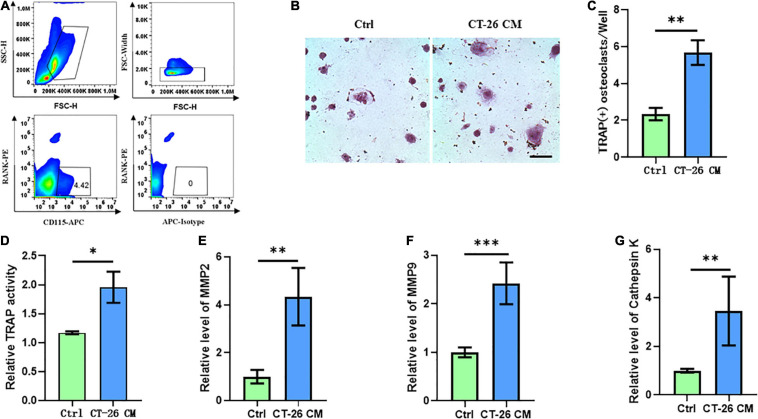
Conditioned medium collected from CRC cell line, CT-26, promotes early osteoclast precursors to differentiate into osteoclasts. **(A)** Gating strategy for sorting out early osteoclast precursors. **(B)** Representative images of TRAP staining demonstrating osteoclast formation of early OCPs under stimulation of CT-26 CM or DMEM (scale bar = 250 μm). **(C)** Quantification of the number of TRAP-positive osteoclasts per well in **(B)**. **(D)** Quantification of relative TRAP activity per well in **(B)**. **(E–G)** The relative expression of osteoclastogenic biomarkers, MMP2, MMP9, and cathepsin K, was determined by real-time PCR. ^∗^*p* < 0.05, ^∗∗^*p* < 0.01, ^∗∗∗^*p* < 0.001.

Few studies focused on the bone metastasis of CRC and the onset of osteolysis. For establishing bone metastasis model in mice, we intratibially injected Balb/c mice with CT-26 CRC cell line. First, early OCPs labeled with CD115-positive and RANK-negative were isolated through FACS (so-called early OCPs). Approximately 4.5% of early OCPs can be sorted out from bone marrow in normal mice (Figure 1A). As expected, during osteoclastogenic induction treated with RANKL and M-CSF for 6 days, early OCPs formed TRAP-positive multinuclear giant cells. Notably, TRAP-staining results suggested higher TRAP activity and more osteoclast number were identified in OCPs after indirect coculture with CT-26 cells (Figures 1B,C). In addition, relative TRAP activity assay confirmed that TRAP activity increased after indirectly coculturing with CT-26 cells (Figure 1D). Consistently, the mRNA levels of osteoclastogenic markers, MMP-2, MMP-9, and cathepsin K, were significantly upregulated in OCPs stimulated with CT-26 CM (Figures 1E–G). These data suggested secreta from CRC cells induce osteoclastogenesis of OCPs.

### miRNA-7062-5p Promotes Osteoclastogenesis of Early OCPs

**FIGURE 2:**
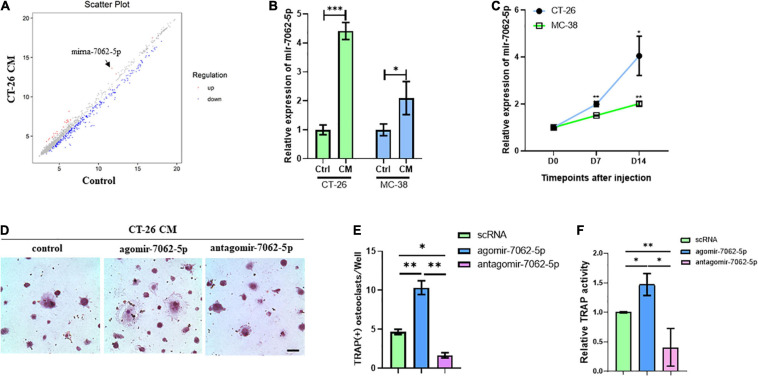
The expression of miRNA-7062-5P upregulates during osteoclastogenesis induced by CT-26 CM and promotes osteoclast formation. **(A)** Scatter plots for microarray assay demonstrated differentially expressed miRNAs in early OCPs treated with CT-26 CM or DMEM. **(B)** The change folders of miRNA-7062-5p level in early OCPs after treatment by CT-26 CM or MC-38 CM compared with the control group. **(C)** The change of miRNA-7062-5p expression in sorted early OCPs at specific timepoints after injection of CT-26 cells or MC-38 cells into tibias. **(D)** Representative images of TRAP staining demonstrating osteoclastogenesis after early OCPs stimulated by RANKL and M-CSF in the presence of CT-26 CM after transfection with controls, agomir-7062-5p, or antagomir-7062-5p (scale bar = 250 μm). **(E)** Quantification of the number of TRAP positive osteoclasts per well in **(D)**. **(F)** Quantification of relative TRAP activity per well in **(D)**. ^∗^*p* < 0.05, ^∗∗^*p* < 0.01, ^∗∗∗^*p* < 0.001.

To assess microRNAs involved in CRC cell–induced osteoclastogenesis, OCPs stimulated with CT-26 CM plus RANKL and M-CSF were collected. Microarray assays revealed that 60 miRNAs were differently expressed during differentiation. Among them, miRNA-7062-5p was the most obviously increased miRNA in CT-26 CM–treated OCPs (Figure 2A). To explore whether miRNA-7062-5p participates in CRC-induced osteoclastogenesis, CM was collected from two CRC cell lines, CT-26 and MC-38 cells, respectively. RT-PCR demonstrated the expression of miRNA-7062-5p upregulated in OCPs after stimulation with both CT-26 CM and MC-38 CM (Figure 2B). Notably, the level of miRNA-7062-5p in primary OCPs increased over time after injection of CT-26 or MC-38 cells intratibially (Figure 2C). These data indicated miRNA-7062-5p positively correlated with osteoclastogenesis and can be induced by secreta derived from CRC cells. To further assess whether miRNA-7062-5p plays a role in regulating osteoclastogenesis in tumor microenvironment, primary OCPs were treated with agomir-7062-5p or antagomir-7062-5p and then stimulated in CT-26 CM, as well as RANKL and M-CSF. TRAP staining and relative TRAP activity results indicated the number of osteoclasts, and TRAP activity significantly increased in the agomir-7062-5p–treated group and decreased in the antagomir-7062-5p–treated group (Figures 2D–F), demonstrating that miRNA-7062-5p directly promoted osteoclastogenesis of OCPs.

### MiRNA-7062-5p Directly Targets GPR65

**FIGURE 3:**
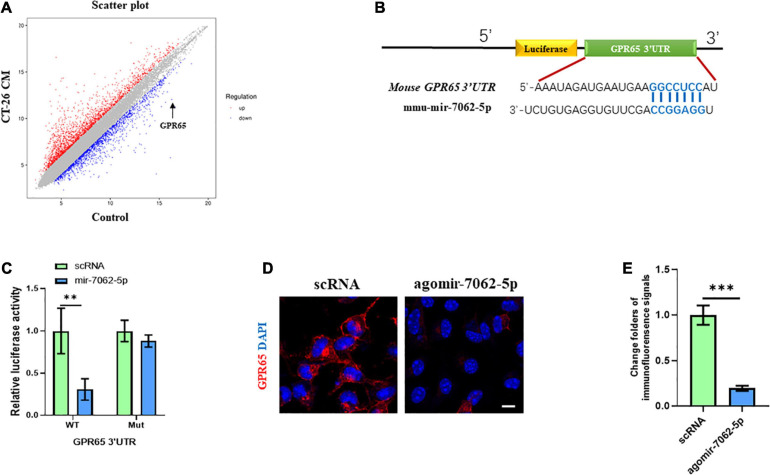
GPR65 is the target gene of miRNA-7062-5p. **(A)** Scatter plots for microarray assay demonstrated differentially expressed mRNAs in early OCPs treated with CT-26 CM or DMEM. **(B)** The complementary sequences of miRNA-7062-5p were discovered in 3′ UTR of GPR65 mRNA using TargetScan. **(C)** Dual-luciferase analysis showed miRNA-70652-5p inversely regulated the luciferase activity of plasmids containing wild-type 3′ UTR of GPR65. **(D)** Immunofluorescence analysis revealed the protein expression of GPR65 in early OPCs after overexpression of miRNA-7062-5p (scale bar = 50 μm). **(E)** Quantification of immunofluorescence intensity in **(D)**. ^∗∗^*p* < 0.01, ^∗∗∗^*p* < 0.001.

To understand the underlying mechanism in miRNA-7062-5p–activated osteoclastogenesis, transcriptomic profiling was analyzed in OCPs treated with CT-26 CM. We detected the differentially expressed genes related to osteoclastogenesis. Interestingly, our results showed the mRNA level of GPR65 downregulated approximately 25 folders (Figure 3A). As it was reported GPR65 negatively regulated osteoclastic activity in osteoporosis (Hikiji et al., 2014), it implied that GPR65 may play an important role in CRC-induced osteoclastogenesis. We next explored whether miRNA-7062-5p can target GPR65. Notably, online analysis tool (TargetScan^1^) predicted that GPR65 could be one potential target gene of miRNA-7062-5p (Figure 3B). Notably, the luciferase activity of 293T cells that were transfected wt 3′ UTR of GPR65 was remarkedly reduced by miRNA-7062-5p, whereas the luciferase activity in group transfection with the mutated-type (mut) 3′ UTR of GPR65 was not affected by miRNA-7062-5p (Figure 3C). In addition, up-regulation of miRNA-7062-5p significantly reduced the protein expression of GPR65 in OCPs; the immunofluorescence signals remarkedly reduced in the miRNA-7062-5p–treated group (Figures 3D,E). Together, these data indicated that GPR65 is the target gene of miRNA-7062-5p. Moreover, overexpression of miRNA-7062-5p can efficiently reduce the protein expression of GPR65 in OCPs.

### miRNA-7062-5p Regulates Osteoclastogenesis of OCPs Dependent on GPR65

**FIGURE 4:**
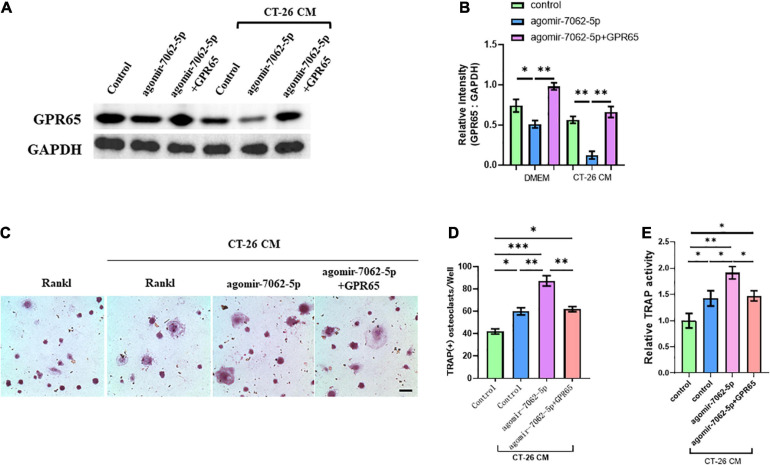
Overexpression of GPR65 reverses the effect of miRNA-7062-5p in early OCPs. **(A)** Western blot analysis demonstrated the protein level of GPR65 in early OCPs after transfection with agomir-7062-5p alone or combination with plasmids containing GPR65 in the presence of CT-26 CM or in normal culture. **(B)** Quantification of relative intensity in **(A)**. **(C)** Representative images of TRAP staining demonstrating osteoclast formation of early OCPs induced by RANKL and M-CSF after transfection with agomir-7062-5p alone or combination with plasmids containing GPR65 in the presence of CT-26 CM (scale bar = 250 μm). **(D)** Quantification of the number of osteoclasts per well in **(C)**. **(E)** Quantification of relative TRAP activity per well in **(C)**. ^∗^*p* < 0.05, ^∗∗^*p* < 0.01, ^∗∗∗^*p* < 0.001.

We demonstrated that GPR65 is the target gene of miRNA-7062-5p, and then we performed rescue experiments to further assess that GPR65 is essential to miRNA-7062-5p–mediated osteoclastogenesis of OCPs. As expected, the protein level of GPR65 downregulated in OCPs after transfection with agomir-7062-5p, whereas the expression of GPR65 was rescued after cotransfection with cDNA3.1(+) containing GPR65 in both normal and CT-26 CM–treated early OCPs (Figures 4A,B). Then, the differentiation of OCPs was investigated. TRAP staining showed that overexpression of GPR65 can decrease the increased number of osteoclasts that were promoted by miRNA-7062-5p transfection in the presence of CT-26 CM (Figures 4C,D). Meanwhile, relative TRAP activity supported the results in TRAP staining; the relative activity was enhanced in agomir-7062-5p–treated group and returned to the level in the control group after overexpression of GPR65 (Figure 4E).

**FIGURE 5:**
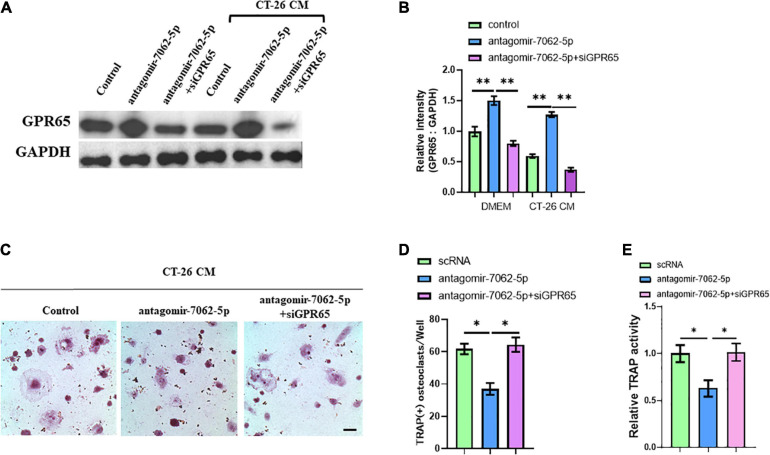
Downregulation of GPR65 reverses the effect caused by inhibiting miRNA-7062-5p. **(A)** Western blot analysis demonstrated the protein level of GPR65 in early OCPs after transfection with antagomir-7062-5p alone or combination with GPR65 siRNA in the presence of CT-26 CM or in normal culture. **(B)** Quantification of relative intensity in **(A)**. **(C)** Representative images of TRAP staining demonstrating osteoclast formation of early OCPs induced by RANKL and M-CSF after transfection with antagomir-7062-5p alone or combination with GPR65 siRNA in the presence of CT-26 CM (scale bar = 250 μm). **(D)** Quantification of the number of osteoclasts per well in **(C)**. **(E)** Quantification of relative TRAP activity per well in **(C)**. ^∗^*p* < 0.05, ^∗∗^*p* < 0.01.

Then, we further proved that miRNA-7062-5p inhibition attenuated osteoclast formation, and this process was dependent on regulating GPR65. OCPs were treated with antagomir-7062-5p or cotreated with siRNA targeting GPR65 in normal medium or CT-26 CM. Western blots showed the protein level of GPR65 in OCPs significantly upregulated after treatment with antagomir-7062-5p, whereas this upregulation can be prevented when cotransfected with siGPR65 (Figures 5A,B). TRAP staining showed miRNA-7062-5p inhibition can decrease the number of osteoclasts, whereas the number was rescued through inhibiting expression of GPR65 in the presence of CT-26 CM (Figures 5C,D). Furthermore, relative TRAP activity demonstrated that TRAP activity was downregulated in antagomir-7062-5p–treated group and recovered after cotransfection with siGPR65 (Figure 5E). These data proved that miRNA-7062-5p stimulated osteoclastogenesis of OCPs through targeting GPR65 in the presence of CT-26 CM.

### GPR65/AMPKα Signaling Inhibited Osteoclastogenesis of OCPs Through Preventing Activation of the JNK Pathway

**FIGURE 6:**
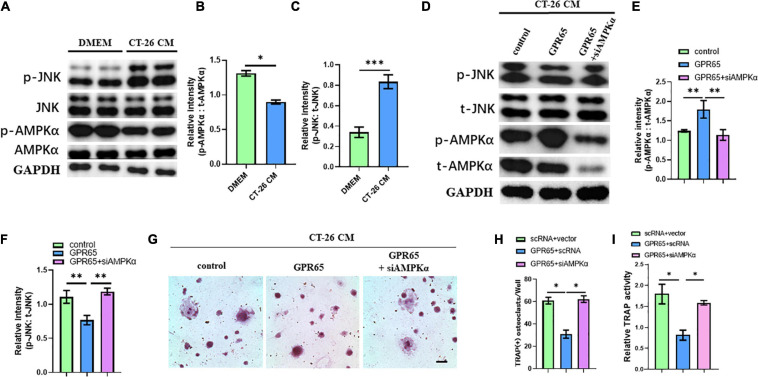
Overexpression of GPR65 stimulates activation of AMPK pathway. **(A)** Western blot analysis showed protein level of activated AMPK and activated JNK compared with their own total proteins in early OCPs cultured in DMEM or CT-26 CM, respectively. **(B)** Quantification of relative intensity of phosphorylation of AMPKα compared with total AMPKαin **(A)**. **(C)** Quantification of relative intensity of phosphorylation of JNK compared with total JNK in **(A)**. **(D)** Western blot analysis showed protein level of activated AMPK and activated JNK compared with their own total proteins in early OCPs after transfection with plasmid containing GPR65 with/without AMPKα siRNA in the presence of CT-26 CM. **(E)** Quantification of relative intensity of phosphorylation of AMPKα compared with total AMPKαin **(D)**. **(F)** Quantification of relative intensity of phosphorylation of JNK compared with total JNK in **(D)**. **(G)** Representative images of TRAP staining demonstrating osteoclast formation of early OCPs induced by RANKL and M-CSF after transfection with plasmids containing GPR65 alone or combination with AMPKα siRNA in the presence of CT-26 CM (scale bar = 250 μm). **(H)** Quantification of relative TRAP activity per well in **(G)**. **(I)** Quantification of the number of osteoclasts per well in **(G)**. ^∗^*p* < 0.05, ^∗∗^*p* < 0.01, ^∗∗∗^*p* < 0.001.

It has been demonstrated that GPR65 was associated with the increased level of cAMP, which can activate AMPK pathway. Interestingly, AMPK pathway may inhibit the activation of the JNK pathway, which is one of the most important pathways involved in osteoclastogenesis. We first detected the protein levels of the JNK pathway and AMPK pathway in CT-26 CM–treated early OCPs compared with normal early OCPs. As expected, the activated JNK protein was upregulated, and the level of activated AMPKα protein was significantly downregulated in early OCPs stimulated by CT-26 CM (Figures 6A–C). To test whether GPR65 regulates AMPK and JNK pathways in OCPs, plasmid containing GPR65 was transfected into OCPs. Interestingly, the activation of AMPKα was significantly enhanced after overexpression of GPR65, and the activation of JNK was prevented, and when cotransfected with AMPKα siRNA, the activation of JNK recovered in early OCPs treated with CT-26 CM (Figures 6D–F). These results showed that GPR65 inhibited activation of JNK through activating phosphorylation of AMPKα in OCPs in CRC microenvironment. To assess whether GPR65 can mediate activation of AMPKα-regulated osteoclastogenic induction in CRC microenvironment, OCPs were induced into osteoclastogenic fate by stimulating with RANKL and M-CSF in the presence of CT-26 CM; meanwhile, plasmid containing GPR65 was transfected or cotransfected with AMPKα siRNA. TRAP staining showed the number of OCs was decreased in GPR65 overexpression group comparing with control group while the number increased after co-transfected with AMPKα siRNA (Figures 6G,H). Consistently, TRAP activity assay revealed overexpression of GPR65 significantly decreased the TRAP activity, but it can be rescued when co-transfected with AMPKα siRNA (Figure 6I). These results demonstrated that GPR65-AMPK pathway regulated the osteoclastogenesis of OCPs in CRC microenvironment.

### Inhibition of miRNA-7062-5p Attenuates Bone Resorption Through Regulating GPR65/AMPK Pathway

**FIGURE 7:**
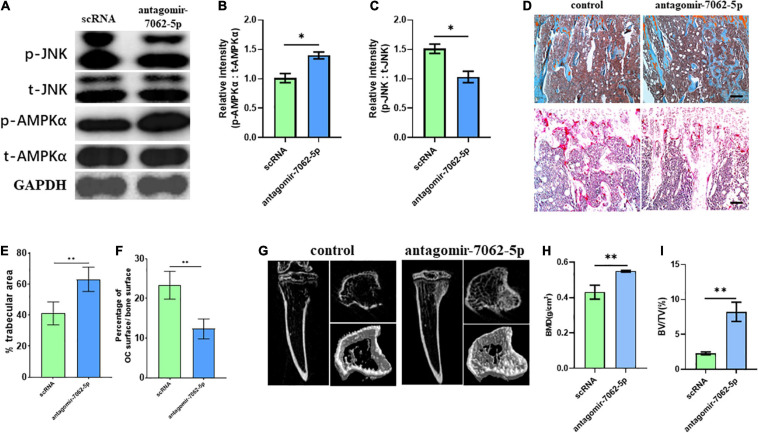
Inhibition of miRNA-7062-5p attenuates bone resorption in bone metastatic model of CT-26 cell line *in vivo*. **(A)** Western blot analysis showed protein level of activated AMPK and JNK compared with its total protein in early OCPs sorted from antagomir-7062-5p–treated bone marrows after injection of CT-26 cells. **(B)** Quantification of relative intensity of phosphorylation of AMPKα compared with total AMPKαin **(A)**. **(C)** Quantification of relative intensity of phosphorylation of JNK compared with total JNK in **(A)**. **(D)** Upper: representative images of safranin O staining *in vivo* after injection with antagomir-7062-5p in bone metastatic model. Down: representative images of TRAP staining *in vivo* after injection with antagomir-7062-5p in bone metastatic model (scale bar = 100 μm). **(E)** Quantification of percentage of trabecular area in upper images in **(D)**. **(F)** Quantification of percentage of TRAP-positive osteoclast surface in total trabecular surface in down images in **(D)**. **(G)** Micro–computed tomography examined the bone destruction after injection with antagomir-7062-5p in bone metastatic model. **(H)** Quantification of bone mineral density in tibias after injection with antagomir-7062-5p in bone metastatic model. **(I)** Quantification of BV/TV in tibias after injection with antagomir-7062-5p in bone metastatic model. ^∗^*p* < 0.05, ^∗∗^*p* < 0.01.

To detect whether anti–miRNA-7062-5p can inhibit osteoclastogenesis *in vivo*, antagomir-7062-5p was administered into tibias after injection of CT-26 cells, and the early OCPs were sorted out. Western blot revealed the activated JNK was inhibited, and the phosphorylated AMPKα was upregulated after treatment with antagomir-7062-5p (Figures 7A–C). Then, we tested whether inhibition of miRNA-7062-5p can prevent bone loss in the bone metastatic model of CRC *in vivo*. Notably, injection of antagomir-7062-5p significantly reduced number of OCs *in vivo* and restored trabecular area (Figures 7D–F). In addition, micro–computed tomography showed that the trabecular area was highly preserved after treatment with antagomir-7062-5p and the bone mineral density and trabecular bone volume fraction (BV/TV) were both improved (Figures 7G–I). These results demonstrated that antagmir-7062-5p can efficiently prevent bone resorption in bone metastasis of CRC.

## Discussion

In osteolytic tumors, bone destruction was positively associated with poor outcome (Powles et al., 1976; Kamalakar et al., 2014; Ma et al., 2018; Jie et al., 2019). It is well known that aberrant activation of osteoclasts contributes to bone resorption after tumor metastasis (Wilson et al., 2008; Das et al., 2011). Targeting osteoclastogenesis can prevent the progression of osteolysis and improve prognosis (Byrne et al., 2019).

Accumulating evidence has revealed that a large number of miRNAs play critical roles in regulating osteoclastogenesis; however, miRNAs involved in bone metastasis were elusive. Ell et al. identified that miR-16 and miR-378 promoted osteolytic bone metastasis in breast cancer and bladder cancer. These two miRNAs may be potential therapeutic targets and biomarkers, indicating that miRNAs could be critical to bone metastasis and tumor progression. As we described, although the incidence of bone metastasis in CRC is relatively less identified, the prognosis is worse. Numerous studies demonstrated that CRC is a kind of cancer that can be markedly regulated by miRNAs (Fang et al., 2012; Ma et al., 2013; Ye et al., 2013; Fu et al., 2014; Li et al., 2015; Xiao et al., 2015; Deng et al., 2016; Feiersinger et al., 2016; Noguchi et al., 2016; Fantini et al., 2017; Boriachek et al., 2018; Kandimalla et al., 2018; Lv et al., 2018). Typically, upregulated miRNAs in cancer promote the cancer, whereas downregulated miRNAs are likely to suppress tumor progression. MiR-155 and miR-503 were two reported miRNAs involved in invasion and metastasis of CRC (Noguchi et al., 2016; Liu et al., 2018). On the other hand, miR-320d, miR-203, miR-144, and miR-99b-5p prevented the proliferation, migration, or invasion in CRC cells (Xiao et al., 2015; Deng et al., 2016; Tang et al., 2019). Although correlation between miR-7062-5p and CRC was not identified in previous studies, we found that the expression of this miRNA can be upregulated in OCPs after stimulation by CRC cells and participate in osteoclast formation, indicating that a novel miRNA, miR-7062-5p, may specifically correlate with bone metastasis from CRC. Our findings together with previous studies revealed the diversity of miRNAs involved in different metastatic sites of CRC.

We here proved GPR65 is the target gene of miR-7062-5p. GPR65 is a member of G protein–coupled receptor family. Recently, the expression of GPR65 was found to be closely associated with intestinal diseases (Tcymbarevich et al., 2019a). Moreover, Tcymbarevich et al. revealed that deficiency in GRP65 in macrophages positively promoted the progression of inflammatory bowel diseases (Tcymbarevich et al., 2019b). As OCPs derive from monocyte/macrophage cell lines, these findings implied that GRP65 may play an important role in regulating physiological process in macrophages. In this study, we identified that overexpression of GPR65 can reverse the osteoclastogenesis induced by miR-7062-5p, indicating that GPR65 plays a role in anti–osteoclast differentiation in bone metastasis of CRC. One study ever reported that TDAG8, another name of GPR65, inhibited osteoclastic activity in osteoporosis (Hikiji et al., 2014), which supports our findings.

Several pathways were reported to be downstream of GPR65, including AP-1 and MEK/ERK (Ryder et al., 2012; Xu et al., 2013), notably, considering close association between GPR65 and MEK/ERK and cAMP, which is linked to the AMPK pathway. In this study, we tested the change of activation of AMPKα and indicated that the overexpression of GPR65 activated phosphorylation of AMPKα. Moreover, miR-7062-5p also downregulated activation of AMPKα, which can be rescued by overexpression of GPR65; thus, we demonstrated that AMPK is downstream of GPR65, and both of them can be regulated by miR-7062-5p. Numerous evidence demonstrated that activation of AMPK negatively regulated osteoclastogenesis (Dong et al., 2018; Tong et al., 2018; Mao et al., 2019). The AMPK pathway can inhibit the activation of the JNK pathway (Lin et al., 2016; Chen S. et al., 2018, Chen X. et al., 2018), which is a key way of stimulating osteoclast formation. In our study, overexpression of GPR65 can inhibit phosphorylation of JNK through activation of AMPKα. Moreover, although overexpression of GPR65 inhibited osteoclast formation, this effect can be rescued by stimulating activation of JNK, indicating that the negative effect of GPR65 on osteoclastogenesis was regulated by activation of JNK.

MiRNAs have been reported to be a promising therapeutic target in tumors. Our results showed that inhibition of miRNA-7062-5p attenuated bone resorption after bone metastasis of CRC, which could be potentially used in the treatment of bone metastasis from CRC.

## Conclusion

**FIGURE 8:**
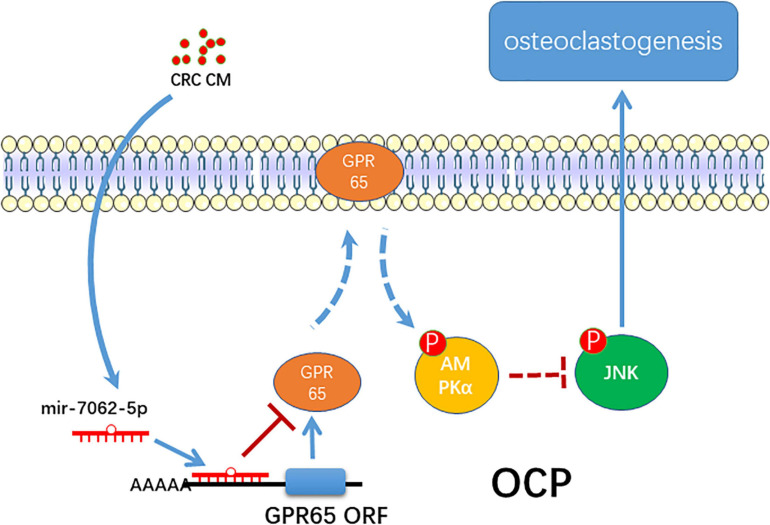
The schematic of miR-7062-5p regulating osteoclastogenesis through targeting GPR65/AMPK pathway.

In summary, the present study proposes a novel role of miRNA-7062-5p in osteoclast formation in bone metastasis of CRC through targeting GPR65, as well as regulating its downstream, AMPK pathway (Figure 8).

## Data Availability Statement

The data presented in the study are deposited in the GEO repository, accession number GSE182097. The link is: https://www.ncbi.nlm.nih.gov/geo/query/acc.cgi?acc=GSE182097.

## Ethics Statement

The animal study was reviewed and approved by the Institutional Animal Care and Use Committee of Daping Hospital at Army Medical University.

## Author Contributions

LC carried out the experiments, analyzed and interpreted the data, and wrote the manuscript. YW analyzed part of data and prepared the manuscript. YW and XL carried out animal experiments and collected samples. XL interpreted part of data and collected samples. LZ and ZW conceived the study, designed the experiments, and wrote the manuscript. All authors read and approved the final manuscript.

## Conflict of Interest

The authors declare that the research was conducted in the absence of any commercial or financial relationships that could be construed as a potential conflict of interest.

## Publisher’s Note

All claims expressed in this article are solely those of the authors and do not necessarily represent those of their affiliated organizations, or those of the publisher, the editors and the reviewers. Any product that may be evaluated in this article, or claim that may be made by its manufacturer, is not guaranteed or endorsed by the publisher.

## Footnotes

1
http://www.targetscan.org

## Abbreviations

CRC: colorectal cancer
TRAP: tartrate-resistant acid phosphatase
CM: conditioned medium
OCPs: osteoclast precursors
M-CSF: macrophage colony stimulating factor
RANKL: receptor activator of nuclear factor-κ B ligand
MMP: matrix metalloproteinase.
